# Modified Apgar Score and Early Physical Parameters in Luxi Black Donkey Foals at Birth: A Prospective Observational Study

**DOI:** 10.3390/vetsci13070669

**Published:** 2026-07-09

**Authors:** Abd Ullah, Liu Bing, Dou Manna, Pengshuai Li, Kang Jiawei, Yu Jie, Muhammad Zahoor Khan, Changfa Wang

**Affiliations:** 1 Dong’e Ejiao Co., Ltd., Shandong Key Laboratory of Gelatine Medicines Research and Development, Dong’e County, Liaocheng 252200, China; 2 College of Agriculture and Biology, Liaocheng University, Liaocheng 252000, China; 3 State Key Laboratory of Animal Nutrition and Feeding, College of Animal Science and Technology, China Agricultural University, Beijing 100193, China

**Keywords:** Apgar score, donkey foal, *Equus asinus*, Luxi Black donkey, neonatal vitality, suckling behavior, thermoregulation

## Abstract

Neonatal assessment in donkey foals is essential for reducing perinatal mortality and improving early management, yet breed-specific data remain scarce, particularly for indigenous Chinese breeds such as the Luxi Black donkey—an economically and genetically valuable breed of Shandong Province for which neonatal vitality has not been previously characterized. This prospective observational study aimed to describe the distribution of a modified four-parameter Apgar score and to summarize early physiological and morphometric parameters in Luxi Black donkey foals at birth. The study further explored whether these early parameters differed across Apgar score categories, with the aim of providing preliminary breed-specific reference data for neonatal assessment in this indigenous donkey breed. Fifty Jennies and the resulting 51 foals were enrolled at the National Black Donkey Breeding Center in Dong’e County, Shandong, China. Within 5 min of delivery, each foal was scored using a modified Apgar system (heart rate, respiratory rate, muscle tone, reflex response; maximum 8), and heart rate, respiratory rate, time to first suckling, rectal temperature, birth weight, body height, body length, chest girth, and abdomen girth were recorded. Foals were grouped according to their observed modified Apgar score categories (8/8, 7/8, 6/8, and 0/8). All 51 foals were included in the descriptive analysis of Apgar score distribution, while the three stillborn foals with an Apgar score of 0/8 were described separately and excluded from statistical comparisons. Physiological and morphometric parameters were compared only among live-born foals with Apgar scores of 8/8, 7/8, and 6/8. Of the 51 foals, 35 (68.63%) scored 8/8, 7 (13.73%) scored 7/8, 6 (11.76%) scored 6/8, and 3 (5.88%) were stillborn. Among live-born foals, respiratory rate (*p* = 0.008), time to first suckling (*p* < 0.001), and rectal temperature (*p* = 0.001) differed significantly among Apgar score categories, whereas heart rate showed no significant difference (*p* = 0.052). Birth weight and morphometric measurements did not differ significantly among live Apgar score categories. These preliminary findings suggest that the modified Apgar score may support early descriptive observation of neonatal status in Luxi Black donkey foals, although further studies with larger cohorts and objective outcome-based measures are needed.

## Simple Summary

Donkeys play a vital role in agriculture and rural livelihoods, particularly in China, where indigenous breeds such as the Luxi Black donkey hold significant economic and cultural value. However, little is known about how to quickly assess the vitality of newborn foals from these native breeds, making it difficult to identify weak neonates that need urgent care. This study addresses that gap by examining 51 Luxi Black donkey foals at birth on a commercial breeding farm in Shandong Province. A simple four-parameter scoring system was applied alongside basic physical and physiological measurements to evaluate each newborn within minutes of delivery. The objective was to generate the first breed-specific reference dataset for Luxi Black donkey foals and to provide a practical, equipment-free tool for early neonatal vitality assessment under field conditions. The aim was to provide the first breed-specific reference data for this indigenous donkey and to offer a practical, equipment-free method that supports better neonatal management, reduces early losses, and contributes to the sustainable conservation of this valuable breed.

## 1. Introduction

Donkeys are important livestock species in many regions, and early neonatal assessment is essential for identifying foals that may require closer observation during the immediate postnatal period [1,2,3]. Donkeys (*Equus asinus*) have historically played an essential role in agriculture, transport, and rural economies worldwide [4,5,6]. In recent years, donkey-related research has increasingly addressed welfare and husbandry, reproductive physiology and management, and conservation of genetic resources, particularly in indigenous and regionally important breeds [7,8]. In China, the donkey is an economically and genetically valuable species that contributes to meat, milk, and traditional medicine industries [6,8,9]. The Luxi Black donkey is an indigenous donkey breed originating from the Luxi region of Shandong Province and is recognized for its uniform black coat, adaptability, and economic value. Optimizing neonatal survival and early-life management is therefore critical for sustainable breeding programs.

The Apgar scoring system, originally developed by Virginia Apgar for the rapid assessment of newborns immediately after delivery [10], provides a simple bedside method for guiding early clinical care and has since become a widely used tool for evaluating neonatal viability in both human and veterinary medicine [10,11]. In its original form, it evaluates five parameters: appearance, pulse, grimace (reflex irritability), activity (muscle tone), and respiration. Each parameter is scored 0 to 2, giving a maximum total score of 10 [10,11]. The system has been adapted for use in pigs [12], lambs [11], calves [11], and puppies [13], and modified versions have been applied to donkey neonates [14]. In the present study, we used a four-parameter modification consisting of heart rate, respiratory rate, muscle tone, and reflex response, each scored from 0 to 2, giving a maximum total score of 8.

Early neonatal adaptation in equids is commonly assessed using behavioral milestones, physiological parameters, and vitality scoring shortly after birth. Previous studies in horse foals and other donkey breeds have shown that respiratory activity, muscle tone, reflex response, suckling behavior, and body temperature are useful indicators of early neonatal condition [14,15,16,17]. However, these parameters may vary among breeds, management systems, and environmental conditions. Although modified Apgar scoring has been applied to some donkey neonates, no breed-specific data are currently available for Luxi Black donkey foals. Therefore, descriptive reference data are needed to support early neonatal assessment and management in this indigenous Chinese breed.

Modified Apgar scoring has previously been described in equine neonates, including horses, mules, and donkey foals [14,18,19,20,21,22,23]. Therefore, the purpose of the present study was not to develop a new scoring system, but to apply an established modified four-parameter Apgar score to Luxi Black donkey foals and describe their early neonatal characteristics. Based on previously used equid neonatal scoring criteria [24,25], equid neonates are classified as normal (score of 7–8), moderately depressed (4–6), or severely depressed (1–3); the lower the score, the more urgent the need for intervention [17]. To date, however, no published data are available for the Luxi Black donkey, an indigenous breed of major economic and cultural importance in eastern China. The primary aim of the present study was to descriptively characterize the distribution of modified Apgar scores and selected early physiological and morphometric parameters in Luxi Black donkey foals at birth. As a secondary exploratory objective, we assessed whether these neonatal parameters differed among observed Apgar score categories. The study was intended to provide preliminary breed-specific descriptive data to support future research and neonatal management.

## 2. Materials and Methods

### 2.1. Ethical Statement and Study Design

**Figure 1:**
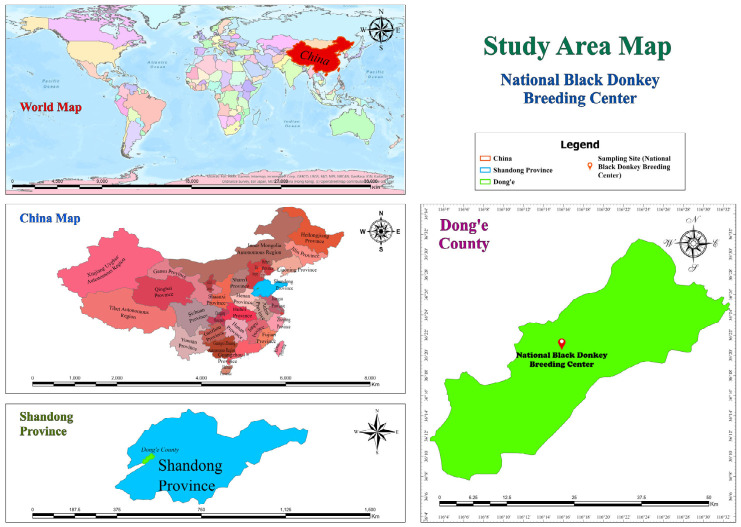
Map of the study area, showing the location of the National Black Donkey Breeding Center in Dong’e County, Shandong Province, eastern China.

The experimental procedures were approved by the Animal Research Ethics Committee of Liaocheng University (AP2025031726). This was a prospective observational study conducted on a single commercial breeding farm. A total of 50 Luxi Black jennies and 51 Luxi Black donkey foals (including one set of twins) were studied. The Luxi Black donkey is one of the most prominent indigenous donkey breeds in China, originating from the Luxi region of Shandong Province in eastern China. It is characterized by its uniform black coat, strong adaptability, and favorable growth performance. The study was conducted at the National Black Donkey Breeding Center, Dong’e County, Shandong Province, China (Figure 1). The study area map shown in Figure 1 was prepared using ArcGIS Desktop 10.8 software (Esri, Redlands, CA, USA).

### 2.2. Delivery Management

All Jennies were maintained under standardized farm management conditions at the National Black Donkey Breeding Center. Pregnant jennies were housed in clean straw-bedded pens during late gestation, defined in this study as the final 30 days before the expected date of parturition, and were transferred to a dedicated foaling area as parturition approached. The expected time of parturition was assessed using insemination records and by monitoring progressive changes in the mammary gland, particularly its enlargement and development during late gestation.

Jennies were fed meadow hay ad libitum and received commercial equine concentrate according to farm practice and NRC energy recommendations [26]. Clean drinking water was available at all times. During the peripartum period, Jennies were regularly monitored by farm staff and veterinarians, and foaling events were attended to ensure timely neonatal assessment. Foaling events occurred between March and April 2026. Parturition time was recorded for each Luxi Black jenny at the time of delivery. Based on the recorded foaling time, parturition events were classified into daytime and nighttime groups. Daytime parturition was defined as occurring from 6:00 AM to 5:59 PM, whereas nighttime parturition was defined as occurring from 6:00 PM to 5:59 AM. During this period, the average temperature was approximately 13.5 °C. The maximum temperature observed was 26 °C, and the minimum temperature was 0 °C. All jennies and foals were included in the descriptive analysis, including stillborn foals, while stillborn foals were excluded from inferential comparisons involving postnatal physiological and morphometric variables. At inclusion, data on maternal age, parity, body height, body length, chest girth, abdomen girth, and colostrum Brix value were recorded. In the case of a twin delivery, both foals were evaluated individually.

### 2.3. Examination and Sampling Procedures

All neonatal assessments were performed within 5 min of birth by the same investigator, who had been trained in foal handling, early neonatal examination, and the modified Apgar scoring procedure before data collection. Minor variability in timing occurred depending on foal handling and stabilization, but all measurements were completed consistently within this 5 min window. A complete physical examination was performed together with a modified four-parameter Apgar score adapted from previous studies [14,18,27]. The score included heart rate, respiratory rate, muscle tone, and reflex response, with each parameter scored from 0 to 2, giving a maximum total score of 8 (Table 1). In addition, morphometric parameters, including birth weight, body height, body length, chest girth, and abdomen girth, were measured. The umbilical cord was manually detached by a veterinarian under hygienic conditions, followed by appropriate antiseptic treatment of the umbilical stump to prevent infection. This procedure was performed as part of routine farm practice and was not an experimental intervention in the present study.

**Figure 2:**
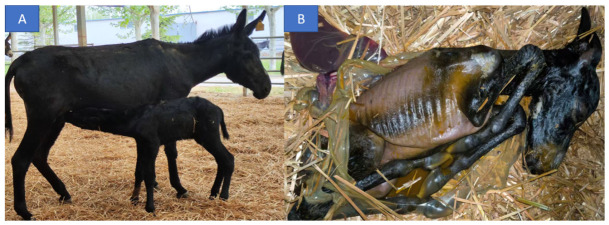
Representative postnatal observations in Luxi Black donkey foals. (**A**) Live singleton foal showing early standing and suckling behavior. (**B**) Stillborn twin foals from one twin delivery were observed during the study.

During the early postnatal examination, foal vitality was assessed based on physical activity, posture, muscle tone, reflex response, suckling behavior, and survival status. Representative images of a healthy singleton foal showing early standing and suckling behavior, and a twin pregnancy in which both twin siblings were stillborn, are presented in Figure 2.

### 2.4. Statistical Analysis

Data were checked for normality using the Shapiro–Wilk test, and the corresponding normality distribution histograms are provided in Supplementary Figure S1. Because some variables deviated from normality and the Apgar score groups were small and unequal in size, non-parametric tests were used. Descriptive statistics are presented as mean ± standard deviation (SD) with minimum–maximum range for continuous variables, and as counts and percentages for categorical variables. Maternal characteristics were summarized descriptively only.

All 51 foals were included in the descriptive analysis of Apgar score distribution. The three stillborn foals with an Apgar score of 0/8 were described separately and excluded from inferential statistical comparisons because postnatal physiological parameters were not applicable. Physiological and morphometric parameters were therefore compared only among live-born foals with Apgar scores of 8/8, 7/8, and 6/8 using the Kruskal–Wallis H test, followed by Dunn’s post hoc test with Bonferroni adjustment when significant differences were detected. To assess whether neonatal vitality differed according to sex, modified Apgar scores and selected physiological and morphometric parameters were compared between live-born colts and fillies using the Mann–Whitney U test, and the results are presented in Supplementary Figures S2 and S3. Sex-wise Apgar score distribution was summarized descriptively as counts and percentages. Statistical analyses were performed using IBM SPSS Statistics v19.0 (IBM Corp., Armonk, NY, USA), and figures were prepared using GraphPad Prism v10.0.2, San Diego, CA, USA). Statistical significance was set at *p* < 0.05.

## 3. Results

### 3.1. Maternal Characteristics of Luxi Black Jennies

Maternal reproductive and morphometric characteristics are summarized in Table 2. The jennies had recorded parities ranging from 1 to 8, and gestation periods ranged from 338 to 367 days. One twin pregnancy was observed during the study period. Based on recorded foaling times, 16 jennies (32.0%) gave birth during the daytime, whereas 34 jennies (68.0%) gave birth during the nighttime. Detailed values for age, parity, gestation period, litter size, colostrum Brix value, and morphometric measurements are provided in Table 2.

### 3.2. Distribution of Apgar Scores in Luxi Black Donkey Foals

A total of 51 Luxi Black donkey foals were evaluated immediately after birth using the modified four-parameter Apgar scoring system. Among these foals, 24/51 (47.1%) were colts, and 27/51 (52.9%) were fillies. Of these, 48 foals survived, whereas 3 foals were stillborn, including one twin pregnancy and one single foal.

**Figure 3:**
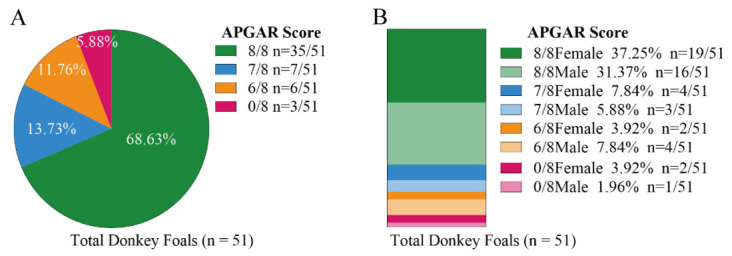
(**A**) Overall distribution of modified Apgar score categories among all 51 Luxi Black donkey foals at birth. (**B**) Sex-wise descriptive distribution of Apgar score categories. Percentages were calculated based on the total number of foals (*n* = 51). Foals with an Apgar score of 0/8 represented stillborn cases.

The overall distribution of Apgar scores is shown in Figure 3A. Among the 51 foals, 35 foals (68.63%) had an Apgar score of 8/8, 7 foals (13.73%) had a score of 7/8, 6 foals (11.76%) had a score of 6/8, and 3 foals (5.88%) had a score of 0/8, representing stillborn cases. The sex-wise distribution of Apgar scores is shown descriptively in Figure 3B. In the 8/8 group, there were 19 fillies (37.25%) and 16 colts (31.37%). In the 7/8 group, there were 4 fillies (7.84%) and 3 colts (5.88%). In the 6/8 group, there were 2 fillies (3.92%) and 4 colts (7.84%). In the 0/8 group, there were 2 fillies (3.92%) and 1 colt (1.96%).

To further clarify the relevance of sex-wise data, selected early physiological and morphometric parameters were compared between live-born colts and fillies and are presented as supplementary data. No significant sex-related differences were observed for heart rate, respiratory rate, and time to first suckling, rectal temperature, birth weight, or morphometric measurements (Supplementary Figures S2 and S3).

### 3.3. Early Physiological Parameters According to Apgar Score

**Figure 4:**
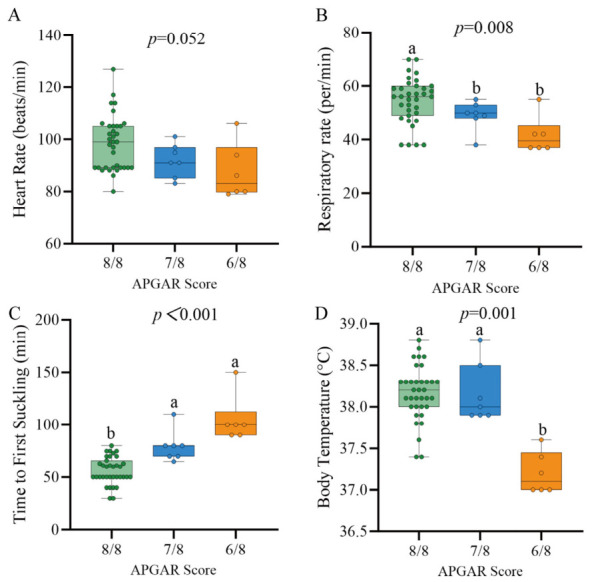
Early physiological parameters among live-born Luxi Black donkey foals according to modified Apgar score category. (**A**) Heart rate, (**B**) respiratory rate, (**C**) time to first suckling, and (**D**) rectal temperature were compared among live-born foals with Apgar scores of 8/8, 7/8, and 6/8. Stillborn foals with an Apgar score of 0/8 were excluded because postnatal physiological measurements were not applicable. Different lowercase letters indicate significant pairwise differences based on Dunn’s post hoc test with Bonferroni adjustment (*p* < 0.05). Panels without lowercase letters indicate no significant pairwise difference.

Early physiological parameters, including heart rate, respiratory rate, time to first suckling, and rectal temperature, were compared only among live-born foals with Apgar scores of 8/8, 7/8, and 6/8. Heart rate showed a decreasing tendency from the 8/8 group to the lower Apgar score groups; however, this difference did not reach statistical significance (*p* = 0.052; Figure 4A).

Respiratory rate differed significantly among live Apgar score categories (*p* = 0.008; Figure 4B). Foals with an Apgar score of 8/8 showed higher respiratory rates, whereas foals with scores of 7/8 and 6/8 showed comparatively lower respiratory rates.

Time to first suckling also differed significantly among live Apgar score categories (*p* < 0.001; Figure 4C). Foals with an Apgar score of 8/8 initiated suckling earlier, while foals with a score of 6/8 required a longer time to suckle.

The suckling reflex was present in all live foals (48/48, 100%), but its strength varied according to Apgar score. Most live foals showed a strong suckling reflex, particularly those with Apgar scores of 8/8 and 7/8. In contrast, foals with an Apgar score of 6/8 showed weak or very weak suckling reflexes.

Body temperature differed significantly among live Apgar score categories (*p* = 0.001; Figure 4D). Foals with Apgar scores of 8/8 and 7/8 maintained relatively stable body temperatures, whereas foals with a score of 6/8 showed lower body temperature values. The 0/8 group represented stillborn foals and was therefore not physiologically comparable with live foals for postnatal functional parameters.

### 3.4. Birth Weight and Morphometric Measurements According to Apgar Score

**Figure 5:**
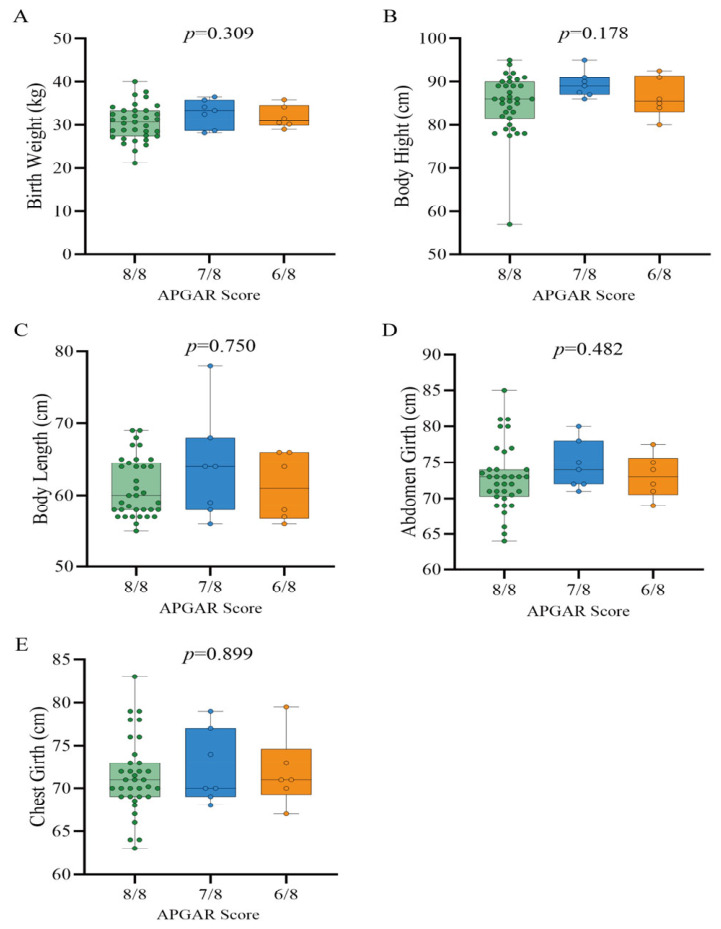
Birth weight and morphometric measurements among live-born Luxi Black donkey foals according to modified Apgar score category. (**A**) Birth weight (**B**), body height (**C**), body length (**D**), abdomen girth (**E**), and chest girth were compared among live-born foals with Apgar scores of 8/8, 7/8, and 6/8 using the Kruskal–Wallis H test. Stillborn foals with an Apgar score of 0/8 were excluded from statistical comparisons and described separately. No significant differences were observed among live Apgar score categories for these parameters; therefore, no lowercase letters are shown.

Birth weight and morphometric measurements were compared only among live-born foals with Apgar scores of 8/8, 7/8, and 6/8. Birth weight did not differ significantly among live Apgar score categories (*p* = 0.309; Figure 5A). The three stillborn foals with an Apgar score of 0/8 were excluded from statistical comparisons and described separately; their mean birth weight was 17.67 ± 3.79 kg.

Similarly, body height, body length, abdomen girth, and chest girth did not differ significantly among live Apgar score categories. Body height showed no significant difference among groups (*p* = 0.178; Figure 5B). Body length (*p* = 0.750; Figure 5C), abdomen girth (*p* = 0.482; Figure 5D), and chest girth (*p* = 0.899; Figure 5E) also showed no significant differences among live Apgar score categories.

### 3.5. Summary of Physiological and Morphometric Parameters by Apgar Score

A summary of early physiological parameters according to live-born Apgar score categories is presented in Table 3. Respiratory rate, time to first suckling, and rectal temperature differed among foals with Apgar scores of 8/8, 7/8, and 6/8, whereas heart rate did not differ significantly. Birth weight and morphometric measurements according to live-born Apgar score categories are summarized in Table 4. Birth weight and external body measurements were generally similar across foals with scores of 8/8, 7/8, and 6/8. The three stillborn foals with an Apgar score of 0/8 were described separately and excluded from statistical comparisons.

## 4. Discussion

To the best of our knowledge, this is the first study to describe the distribution of a modified four-parameter Apgar score and early physiological and morphometric parameters in Luxi Black donkey foals at birth, an indigenous breed of major economic and genetic value in eastern China. The present study should be interpreted as a descriptive characterization of early neonatal parameters rather than as a validation of the modified Apgar score. Although respiratory rate, rectal temperature, and time to first suckling differed among live Apgar score categories, the study did not assess long-term survival, morbidity, passive transfer status, or treatment requirement. Therefore, further studies including objective clinical outcomes are required before the prognostic or clinical decision-making value of the score can be established.

### 4.1. Distribution of Apgar Scores and Breed-Specific Considerations

Most Luxi Black donkey foals (68.63%) scored 8/8, with progressively smaller proportions in the 7/8, 6/8, and 0/8 categories. This distribution suggests that most foals born under the management conditions of the present breeding center showed a favorable early neonatal status. Similar predominance of high Apgar scores has been reported in Amiata donkey foals [14] and in other equid neonates, including horse and mule foals [21,22], suggesting that high early vitality scores are commonly observed in uncomplicated deliveries. The three foals with an Apgar score of 0/8 were stillborn and were therefore described separately rather than included in inferential comparisons among live-born foals. This distinction is important because Apgar scoring primarily reflects postnatal adaptation in live neonates, whereas stillborn foals receive a score of zero. After all, all score components are absent. The 5.88% stillbirth rate observed in the present study is broadly comparable with previous reports from managed donkey populations [14,27]. However, the occurrence of stillbirth may be influenced by several factors, including breed, parity, gestational conditions, management, and pregnancy type. Therefore, the Apgar score distribution in the present study should be interpreted as preliminary descriptive information for Luxi Black donkey foals.

### 4.2. Respiratory Adaptation Across Apgar Score Categories

Respiratory rate, rectal temperature, and time to first suckling differed among live-born foals with Apgar scores of 8/8, 7/8, and 6/8. These observed differences suggest that foals with higher Apgar scores showed more stable early physiological profiles after birth. Respiratory rate differed among Apgar score categories (*p* = 0.008), with 8/8 foals showing values (mean 56 breaths/min) within the published reference range for healthy donkey neonates (20–99 breaths/min) [14,17], and 6/8 foals showing lower values (mean 47 breaths/min). However, because respiratory rate was included as a component of the modified Apgar score, this difference was, by definition, partly expected and should not be interpreted as independent validation of the scoring system.

The transition from fetal to neonatal life requires rapid lung fluid clearance, alveolar expansion, establishment of functional residual capacity, and a switch from placental to pulmonary gas exchange [17,18]. Previous studies have indicated that poor early respiratory responses may be related to delayed catecholamine response, incomplete lung fluid clearance, residual peripartum asphyxia, or central nervous system depression [14,18,24,28,29,30,31]. However, these mechanisms were not directly assessed in the present study because blood gas analysis, lactate concentration, oxygenation status, and specific markers of peripartum asphyxia were not measured. Therefore, lower respiratory activity in lower Apgar score categories should be interpreted cautiously as a descriptive finding rather than as mechanistic evidence. Similar respiratory patterns have been reported in Amiata donkey foals [14], suggesting that early respiratory adaptation may show comparable trends across donkey breeds.

### 4.3. Heart Rate and Cardiovascular Transition Across Apgar Score Categories

Heart rate decreased progressively with lower Apgar score (98 → 92 → 89 beats/min) but did not reach statistical significance (*p* = 0.052). Several biological and statistical factors deserve consideration. First, the immediate postnatal heart rate in equid neonates is intrinsically variable; values from 33 to 182 beats/min have been recorded within the first 5 min in donkey foals [14,17]. Heart rate may be influenced by transient sympathetic activation, ongoing physical effort during early attempts at sternal recumbency and standing, cardiovascular transition after birth, changes in pulmonary vascular resistance, and ambient temperature [17,28,30]. This wide physiological range may increase within-group variation and reduce the ability to detect differences among Apgar score categories, particularly with the present sample size.

Second, although bradycardia is considered an important cardiovascular sign in compromised foals [24,25], previous studies have also indicated that severely compromised neonates may show variable cardiovascular responses, including tachycardia related to sympathetic activation or metabolic disturbance. Such variation may obscure a strictly linear relationship between Apgar score category and heart rate. Importantly, heart rate was also included as one component of the modified Apgar score; therefore, the observed borderline trend should not be interpreted as independent validation of the scoring system. Because the difference was not statistically significant, heart rate should be considered only as a supportive physiological parameter and interpreted together with other neonatal indicators rather than used alone as an independent indicator of vitality [24,28,29].

### 4.4. Thermoregulatory Vulnerability of Low-Scoring Foals

Rectal temperature was significantly lower in 6/8 foals (mean 37.2 °C) than in 7/8 or 8/8 foals (mean 38.2 °C; *p* = 0.001). Equid neonates are particularly vulnerable to hypothermia because they possess limited brown adipose tissue and rely mainly on shivering thermogenesis, vasomotor control, and behavioral mechanisms, such as standing, maternal contact, and prompt colostrum intake, to maintain core temperature [17,30,31]. The mean ambient temperature during this study (13.5 °C; minimum 0 °C) may have increased the thermal challenge for newborn foals.

The lower rectal temperature observed in 6/8 foals may be related to delayed early adaptation, delayed suckling, or reduced early activity; however, these mechanisms were not directly investigated in the present study. Therefore, possible explanations such as reduced shivering, impaired peripheral perfusion, or delayed caloric intake should be considered hypotheses rather than confirmed mechanisms. Previous studies have reported that hypothermia in foals may be associated with hypoglycemia, reduced gut motility, and impaired IgG absorption [17,28]. In the present study, however, blood glucose, IgG concentration, and metabolic status were not assessed. Thus, rectal temperature differences should be interpreted cautiously as descriptive findings that may indicate the need for closer early monitoring in lower-scoring foals.

### 4.5. Time to First Suckling as a Behavioral Integrator of Neonatal Vitality

The strongest separation among groups was observed for time to first suckling (*p* < 0.001; 56 min in 8/8, 79 min in 7/8, and 105 min in 6/8 foals). Foals with lower Apgar scores, particularly those scoring 6/8, showed delayed initiation of suckling, which may reflect slower early behavioral or neuromuscular adaptation. This observation is descriptive and does not imply clinical dysfunction.

Suckling is not a single reflex but an integrated behavior requiring intact brainstem function, adequate olfactory and tactile processing, sufficient muscle tone to stand and orient toward the udder, and coordination of jaw, tongue and pharyngeal musculature [18,28,29]. Previous studies have emphasized the importance of early colostrum intake because equid foals depend on enteral absorption of colostral IgG, with greatest absorptive efficiency during the early postnatal period [18,27,31]. However, IgG concentration and passive transfer status were not assessed in the present study. Therefore, the observed delay in suckling among 6/8 foals should not be interpreted as evidence of failure of passive transfer or neonatal sepsis. Instead, delayed suckling should be interpreted cautiously as an early behavioral finding that may indicate the need for closer monitoring and clinical assessment, in line with established equine neonatal care recommendations [17,28].

### 4.6. Birth Weight, Stillbirth, and the Contribution of Twin Pregnancy

Birth weight was similar among live-born foals with Apgar scores of 8/8, 7/8, and 6/8. The three stillborn foals with an Apgar score of 0/8 were descriptively lighter (17.7 kg) and were considered separately because they represented a biologically distinct outcome. The lower birth weight observed in stillborn foals may reflect compromised fetal development or reduced perinatal viability; however, because only three stillborn cases were recorded, this finding should be interpreted descriptively and confirmed in larger studies. A single twin pregnancy resulted in both Luxi Black donkey foals being stillborn, illustrating the elevated perinatal mortality risk associated with twin pregnancies, in which the diffuse epitheliochorial placenta is poorly adapted to support two concurrent fetuses.

Two of the three stillborn foals in the present cohort originated from a single twin pregnancy in which both twin siblings died, while the third stillborn foal was from a singleton parturition. The loss of both twin fetuses provides a striking illustration of the reproductive disadvantage of twinning in donkeys: as in horses, the diffuse epitheliochorial placenta in donkeys distributes microcotyledons across nearly the entire endometrial surface and cannot effectively partition placental support between two conceptuses, leading to severe placental insufficiency, intrauterine growth restriction of one or both fetuses, and frequently the death of both twins through abortion, stillbirth, or early neonatal failure [17,30,32]. However, because only one twin pregnancy was observed in the present study, its contribution to stillbirth and low birth weight should be interpreted cautiously. Future studies with larger datasets are needed to evaluate the effects of twin pregnancy, parity, placental factors, and gestational conditions on stillbirth risk in Luxi Black donkeys.

### 4.7. Independence of Most Morphometric Parameters from the Apgar Score

Body height, body length, chest girth, and abdomen girth did not differ among the live Apgar score groups (all *p* > 0.05). This is biologically coherent: external linear dimensions are determined predominantly by genotype, sex, and gestation length and provide a measure of structural rather than functional maturity [31,32]. Morphometric parameters mainly describe structural body size and breed conformation, whereas Apgar scoring reflects immediate postnatal physiological and behavioral responses.

Therefore, the lack of significant differences in morphometric measurements among live Apgar score categories suggests that body size alone may not reflect early neonatal status. In contrast, early functional observations, such as respiratory activity, rectal temperature, and time to first suckling, may be more useful for describing early neonatal adaptation. However, these findings should be interpreted cautiously, and further studies are needed to determine their clinical relevance.

### 4.8. Ancillary Observations: Sex Distribution and Timing of Parturition

Foal sex and time of foaling were evaluated as exploratory perinatal factors. In the present study, 24/51 foals were colts and 27/51 were fillies. Modified Apgar scores and selected early physiological and morphometric parameters were compared between live-born colts and fillies, and no significant sex-related differences were observed.

Nighttime parturition predominated (68.0%), consistent with the well-documented chronobiology of equid foaling, which is regulated by maternal melatonin, prolactin, and cortisol rhythms favoring delivery during periods of reduced disturbance [32,33]. Foaling time was summarized descriptively as daytime or nighttime. A higher proportion of foalings occurred during the nighttime than during the daytime. However, the present study did not formally evaluate the effect of foaling time on Apgar score, physiological parameters, or neonatal outcomes. Therefore, nighttime foaling should be interpreted as a descriptive management observation only. Further studies with larger sample sizes and planned analysis of perinatal risk factors are required to determine whether foaling time influences neonatal vitality in Luxi Black donkey foals.

### 4.9. Practical and Clinical Implications

The findings of the present study suggest that the modified four-parameter Apgar score may be a simple practical method for describing early neonatal status in Luxi Black donkey foals. The score can be applied within 5 min of delivery without specialized equipment and may support early observation of foals during the immediate postnatal period. However, the present study did not evaluate treatment response, passive transfer status, morbidity, or long-term survival; therefore, specific clinical intervention thresholds cannot be established from these data.

Foals with higher Apgar scores appeared to show more favorable early physiological profiles after birth, particularly in respiratory rate, rectal temperature, and early suckling behavior. Foals with lower live Apgar scores, particularly those scoring 6/8, showed delayed time to first suckling and lower respiratory rate and rectal temperature compared with higher-scoring foals. These findings suggest that lower-scoring foals may require closer postnatal monitoring. However, practical management decisions, such as assisted nursing, thermal support, colostrum supplementation, IgG assessment, fluid therapy, or intensive veterinary care, should be based on individual clinical evaluation and established equine neonatal guidelines rather than on the present Apgar score data alone [17,24,28]. Overall, the modified Apgar score may support early observation of neonatal vitality in Luxi Black donkey foals, but further studies including objective clinical outcomes are required before breed-specific management cut-off values can be proposed.

### 4.10. Strengths, Limitations, and Future Directions

Strengths of the present study include its prospective design, standardized assessment timing within 5 min of birth, and the characterization of an indigenous breed for which no neonatal reference data previously existed. Several limitations should nevertheless be acknowledged. First, the study population consisted mainly of high-scoring, apparently healthy live-born foals, and no live-born foals were observed in the Apgar score range of 1–5. Therefore, the present study cannot evaluate the modified Apgar score across its full intended clinical range or validate previously proposed vitality categories.

Second, the lower Apgar score categories were small, limiting statistical power and precluding firm conclusions about the borderline heart rate trend (*p* = 0.052). The three stillborn foals with an Apgar score of 0/8 were described separately and excluded from inferential statistical comparisons; therefore, conclusions are based on comparisons among live-born foals only. In addition, two of the three stillborn foals originated from the same twin pregnancy, meaning that a substantial proportion of adverse outcomes was related to a single reproductive event. Therefore, the stillbirth findings should be interpreted descriptively and confirmed in larger studies.

Third, assessment was restricted to physical and behavioral parameters. Blood gas analysis, hematology, biochemistry, IgG measurement to assess passive transfer status, and lactate, a sensitive marker of peripartum asphyxia in foals [33], were not collected and would strengthen future mechanistic interpretation. Fourth, all foalings occurred at a single breeding center under standardized assisted-delivery management, and the results may not transfer directly to extensive or smallholder systems with different husbandry conditions.

All live-born foals were monitored for 7 days after birth, and no mortality was observed during this period. However, because all live-born foals survived during the short follow-up period, the present study could not determine whether different Apgar score categories predict neonatal mortality or longer-term clinical outcomes.

Future multicenter studies in Luxi Black and other Chinese indigenous donkey breeds, combining the modified Apgar score with hematobiochemical profiling, IgG status, blood gas analysis, and longitudinal growth and morbidity follow-up, are required to further evaluate the clinical relevance and predictive value of the score beyond the immediate postnatal period.

## 5. Conclusions

This study provides the first preliminary breed-specific descriptive data on the distribution of modified Apgar scores, early physiological parameters, and morphometric measurements in Luxi Black donkey foals at birth. Among 51 foals, 68.63% scored 8/8, while respiratory rate, time to first suckling, and rectal temperature differed significantly across score groups, indicating descriptive differences among Apgar scores. These findings should be interpreted as descriptive differences in early neonatal parameters rather than as validation of the modified Apgar score or evidence of its predictive performance. Heart rate showed a non-significant decreasing trend (*p* = 0.052), and external morphometric parameters did not differ among live-born Apgar score categories. Birth weight did not differ significantly among live-born foals with Apgar scores of 8/8, 7/8, and 6/8. The three stillborn foals with an Apgar score of 0/8 were described separately, and their lower birth weight should be interpreted cautiously because only three stillborn cases were recorded, and two originated from the same twin pregnancy.

Overall, the modified Apgar score may support early observation and descriptive assessment of neonatal status in Luxi Black donkey foals. However, the present study did not evaluate diagnostic performance, treatment response, passive transfer status, morbidity, or long-term survival; therefore, the score should not be considered a validated screening or clinical decision-making tool at this stage. Future multicenter studies incorporating hematobiochemical profiling, IgG status, blood gas analysis, treatment response, and longitudinal follow-up are required to evaluate the clinical relevance and predictive value of the modified Apgar score in indigenous Chinese donkey breeds.

## Figures and Tables

**Table 1:** Modified Apgar scoring system used to assess neonatal vitality in Luxi Black donkey foals (adapted from [14,27]).

Parameters	0 Points	1 Point	2 Points
Heart rate/pulse quality	Undetectable	<60 beats/min, irregular	>60 beats/min, regular
Respiratory rate/pattern	Undetectable	Slow/irregular <40/breaths/min	>40 breaths/min, regular
Muscle tone	Limp/absent	Lateral recumbency, some tone, some flexion of limb	Sternal recumbency
Reflex response (irritability)	Unresponsive (no reaction to touch or stimuli)	Grimace, mild rejection (slight response to touch)	Cough or sneeze (strong response to touch)

**Table 2:** Descriptive statistics of maternal reproductive and morphometric characteristics in Luxi Black jennies (*n* = 50).

Parameter	N	Minimum	Maximum	Mean ± SD
Age (years)	50	3	14	6.54 ± 2.67
Parity (n)	50	1	8	2.92 ± 1.79
Gestation length (days)	50	338	367	351.2 ± 6.6
Litter size	50	1.00	2.00	1.02 ± 0.14
Colostrum Brix value (%)	50	12.00	32.00	21.35 ± 3.91
Body height (cm)	50	120.00	155.00	139.56 ± 6.03
Body length (cm)	50	119.00	150.00	135.07 ± 6.93
Chest girth (cm)	50	140.00	163.00	151.98 ± 6.90
Abdomen girth (cm)	50	149.00	206.50	179.45 ± 13.33

**Table 3:** Early physiological parameters of live-born Luxi Black donkey foals according to modified Apgar score category.

Parameter	Apgar 8/8 (*n* = 35)			Apgar 7/8 (*n* = 7)			Apgar 6/8 (*n* = 6)		
	Mean ± SD	Min	Max	Mean ± SD	Min	Max	Mean ± SD	Min	Max
Heart rate (beats/min)	98.37 ± 10.52	80	127	91.86 ± 6.41	83	101	88.67 ± 10.07	79	106
Respiratory rate (breaths/min)	56.11 ± 6.24	45	70	50.86 ± 2.41	48	55	47.00 ± 7.85	42	59
Time to first suckling (min)	56.09 ± 12.91	30	80	79.29 ± 14.84	65	110	105.00 ± 22.58	90	150
Body Temperature (°C)	38.16 ± 0.33	37.4	38.8	38.16 ± 0.36	37.9	38.8	37.20 ± 0.25	37.0	37.6

Continuous variables for each group are presented as mean ± SD (minimum–maximum) using descriptive statistics in SPSS. Stillborn foals with an Apgar score of 0/8 were excluded because postnatal physiological measurements were not applicable.

**Table 4:** Birth weight and morphometric measurements of live-born Luxi Black donkey foals according to modified Apgar score category.

Parameter	Apgar 8/8 (*n* = 35)			Apgar 7/8 (*n* = 7)			Apgar 6/8 (*n* = 6)		
	Mean ± SD	Min	Max	Mean ± SD	Min	Max	Mean ± SD	Min	Max
Birth weight (kg)	30.50 ± 4.13	20	40	32.76 ± 3.24	28.20	36.50	31.88 ± 2.62	29	35.85
Body height (cm)	85.00 ± 6.91	57	95	89.36 ± 3.04	86	96	86.42 ± 4.63	80	92.50
Body length (cm)	61.04 ± 4.11	55	69	63.86 ± 7.49	56	78	61.17 ± 4.67	56	66
Chest girth (cm)	71.38 ± 4.46	63	83	72.43 ± 4.28	68	79	71.92 ± 4.20	67	79.50
Abdomen girth (cm)	72.91 ± 4.62	64	85	74.57 ± 3.36	71	80	73.08 ± 3.04	69	77.50

Values are presented for live-born foals only as mean ± SD, minimum, and maximum. Stillborn foals with an Apgar score of 0/8 were excluded from statistical comparisons and described separately.

## Data Availability

The original contributions presented in this study are included in the article/Supplementary Material. Further inquiries can be directed to the corresponding authors.

## Supplementary Materials

The following supporting information can be downloaded at https://www.mdpi.com/article/10.3390/vetsci13070669/s1. Supplementary Figure S1: normality distribution histograms; Supplementary Figures S2 and S3: sex-specific distributions of modified Apgar scores and selected physiological and morphometric parameters.
