# Supplementary material for: Modified Apgar Score and Early Physical Parameters in Luxi Black Donkey Foals at Birth: A Prospective Observational Study
Source: Vet Sci. 2026 Jul 9;13(7):669. doi: 10.3390/vetsci13070669 (PMC13431603; doi:10.3390/vetsci13070669)
Supplement: Supplementary file 1 [file vetsci-13-00669-s001.zip › vetsci-4367467-supplementary.pdf]

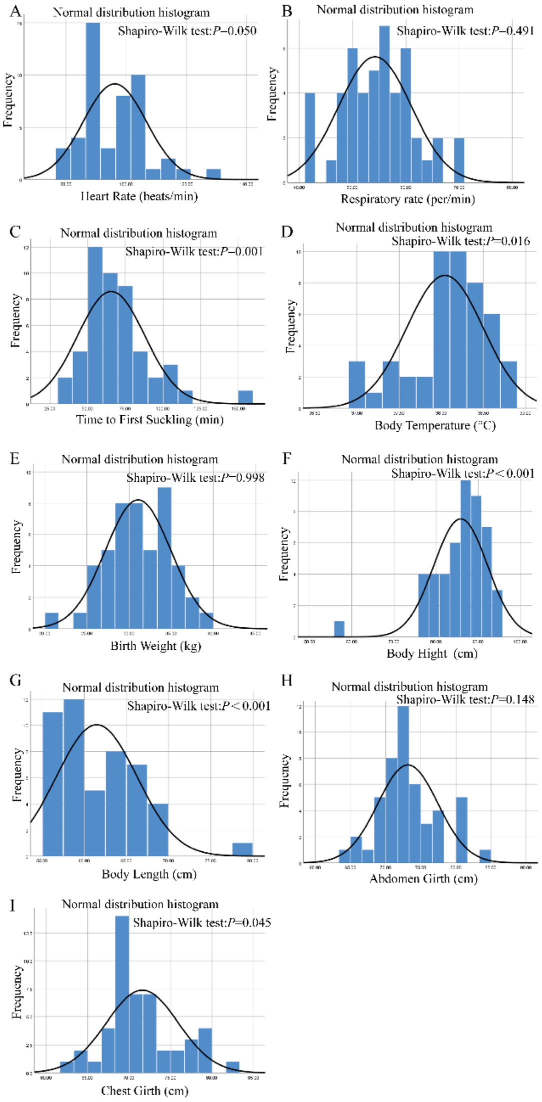

Supplementary Figure S1. Normality distribution histograms of early physiological and morphometric parameters in live-born Luxi Black donkey foals. Histograms with fitted normal curves are shown for (A) heart rate, (B) respiratory rate, (C) time to first suckling, (D) rectal temperature, (E) birth weight, (F) body height, (G) body length, (H) abdomen girth, and (I) chest girth. Normality was assessed using the Shapiro–Wilk test, with $p < 0.05$ indicating deviation from normal distribution.

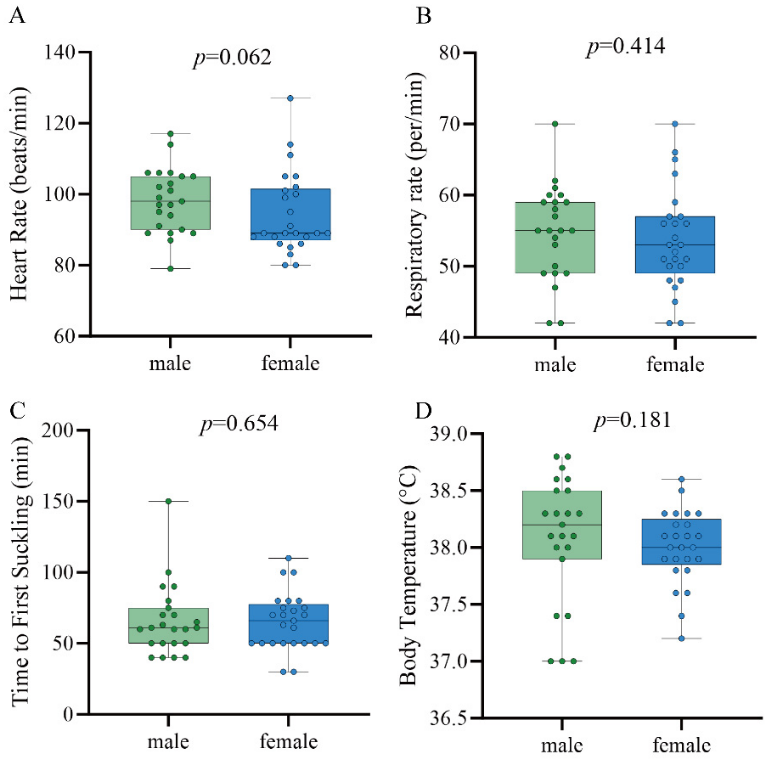

Supplementary Figure S2. Sex-wise comparison of early physiological parameters in live-born Luxi Black donkey foals. (A) Heart rate, (B) respiratory rate, (C) time to first suckling, and (D) rectal temperature were compared between colts and fillies. No significant differences were observed between sexes ($p > 0.05$).

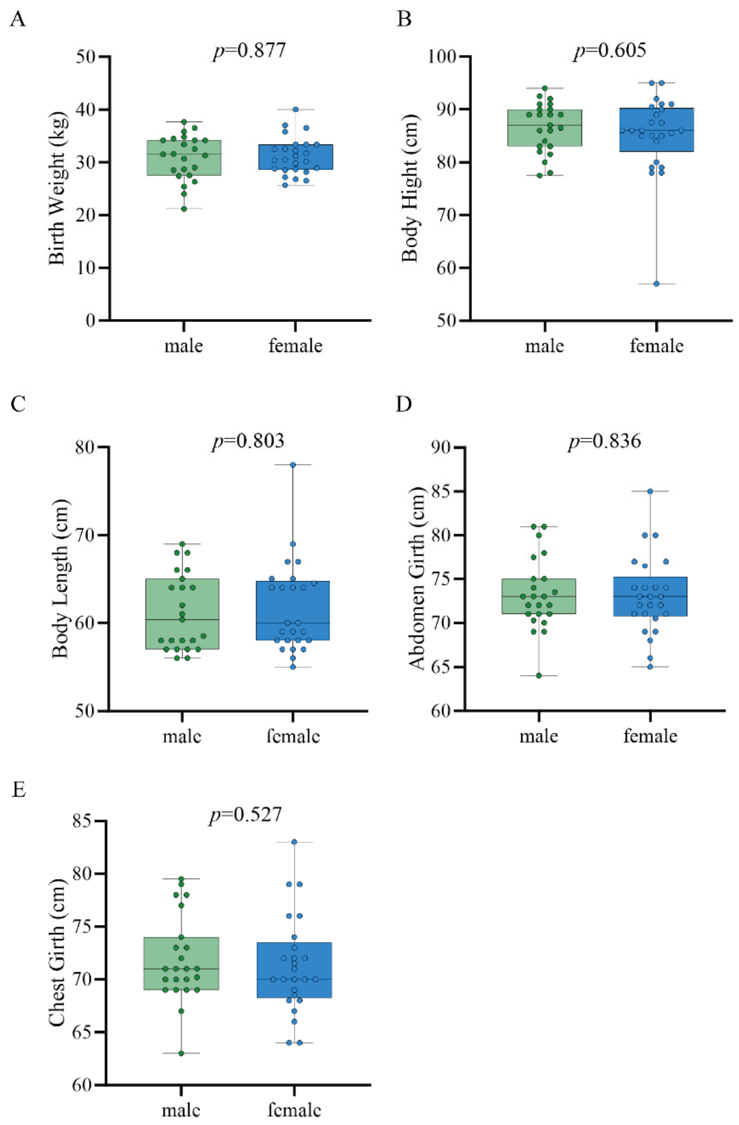

Supplementary Figure S3. Sex-wise comparison of birth weight and morphometric measurements in live-born Luxi Black donkey foals. (A) Birth weight, (B) body height, (C) body length, (D) abdomen girth, and (E) chest girth were compared between colts and fillies. No significant differences were observed between sexes ($p > 0.05$).
